# Diphtheria, Tetanus, and Pertussis Immunity in Indian Adults and Immunogenicity of Td Vaccine

**DOI:** 10.5402/2011/745868

**Published:** 2011-12-28

**Authors:** Prasad S. Kulkarni, Sidram K. Raut, Sanjay P. Dhorje, Prajakt J. Barde, Girish Koli, Suresh S. Jadhav

**Affiliations:** ^1^Clinical Department, Serum Institute of India Ltd., 212/2, Hadapsar, Pune 411028, India; ^2^Medical Department, Serum Institute of India Research Foundation, Pune 411028, India; ^3^Quality Control Department, Serum Institute of India Ltd., 212/2, Hadapsar, Pune 411028, India

## Abstract

Rise of diphtheria cases in adults is a cause of concern worldwide. Pertussis is also now affecting adults. We assessed serum levels of tetanus, diphtheria and pertussis antibodies in 62 adults in Pune, India, who had missed their primary immunization. All adults were then given three doses of tetanus-diphtheria (Td) vaccine at 0, 1, and 6 months. All adults were immune to tetanus but 78% had long-term protection. For diphtheria, 88% were protected but only 9% had long term immunity. Only 60% were immune to pertussis. After three doses of the vaccine, long term immunity to both tetanus and diphtheria increased to 87% and 97%, respectively (*P* < 0.05). Geometric mean titres (GMT) of both antibodies also increased significantly. The vaccine caused minor local reactions and mild fever in a few subjects. There is need of three doses of Td vaccination in those Indian adults, who missed their primary immunization. Susceptibility to pertussis also needs to be further explored.

## 1. Introduction

In the 1990s, a large epidemic of diphtheria began in Russia and subsequently spread to the Newly Independent States (NIS) of the former Soviet Union. About two-thirds of the reported cases occurred among persons ≥15 years of age. In Ukraine too, at the peak of the epidemic in 1995, more than 80% cases were reported in the same age group [1–4]. In fact, serologic studies in the 1980s from these countries had suggested that >50% of adults were susceptible to diphtheria [5, 6]. Since then, diphtheria immunity among adults became an important issue.

Tetanus too remains an important public health problem in many parts of the world, particularly in the tropical developing countries. In 2008, the total number of deaths caused by tetanus worldwide was estimated to be more than 61,000 [7].

In India, DTP vaccine was introduced in routine immunization in 1978, resulting in substantial decline in incidence in the pediatric populations. The effect was a shift of the infection to the older age groups. In 1998, around 65% of cases occurred above 3 years of age. The age shift justified the need of booster diphtheria immunization [8].

Therefore, the World Health Organization (WHO) recommends three doses of diphtheria toxoid-containing vaccination of adults who may have not been primed previously neither by natural infection nor by vaccination [9].

Pertussis is generally considered as a childhood disease but was well documented in adults during the twentieth century [10–12]. Recently, in the United States, there has been an increase in pertussis among adolescents and adults [13, 14]. In India, there are no reports of pertussis in adults yet but chances are that these cases are not detected and the susceptibility is also not known.

In the present study, we assessed the diphtheria, pertussis, and tetanus immunity in adult individuals who missed primary DTP immunization. We also assessed effect of three-dose schedule of a tetanus-diphtheria (Td) vaccine in this population. Td vaccine is not combined with whole cell pertussis because of higher reactions in this age group [15].

Td vaccine manufactured by Serum Institute of India Ltd (SIIL) is licensed in India. It is also prequalified by WHO for the sale to the United Nations agencies since 1995. The vaccine is safe and immunogenic [16]. Millions of doses of this vaccine have been used worldwide.

## 2. Materials and Methods

### 2.1. Setting

The study was conducted at the clinic of Serum Institute of India Research Foundation (SIIRF), Pune, after the Ethics Committee approval. Adult employees of the Poonawalla group of companies were enrolled after taking written informed consent. The study was conducted between May and November 2007.

### 2.2. Study Procedures

On day 0, subjects were screened for eligibility and then enrolled in the study. Blood samples were collected for baseline serological status. Three doses of the Td vaccine were given on 0, 1, and 6 months to all the subjects. They were asked to record adverse events in diary cards. On each visit, medical history was asked for adverse events and concomitant medications. Physical examination was performed. One month after the third dose, the second blood sample was taken for serology.

### 2.3. Study Population

The Expanded Programme on Immunization (EPI) was initiated in India in 1978 which included DTP and DT vaccines. Hence, healthy adults of age 30 years to 65 years (born before 1978), who gave consent and who had not received DTP or DT vaccines in the past, were selected. Subjects with pregnancy and lactation or any medical disorder or allergy were excluded. Contraindications for the subsequent doses were any serious adverse event (SAE) following the previous dose.

### 2.4. Study Vaccine

Three doses of 0.5 mL of Td vaccine (Batch Number A-404-B; Expiry Date: February, 2009) manufactured by Serum Institute of India Ltd (SIIL), Pune, were administered in a schedule of 0, 1, and 6 months. Each single 0.5 mL dose contains ≤5 Lf (≥2 IU) of diphtheria toxoid, ≥5 Lf (≥40 IU) of tetanus toxoid, adsorbed on ≥1.5 mg of aluminum Phosphate (AlPO_4_). 0.01% thiomersal was used as preservative. The vaccine was injected in the deltoid muscle. The vaccines were transported and stored at 2–8°C.

### 2.5. Serology

Before vaccination, antidiphtheria, antitetanus and antipertussis IgG antibody levels were measured. Postvaccination antidiphtheria and antitetanus antibodies were assessed. The testing was done at the Quality Control Department, SIIL by ELISA kits of Virion/Serion ELISA Classic (Germany). For diphtheria and tetanus, titres ≥0.1 IU/mL indicate safe protection, while >1.0 IU/mL indicate long-term protection. Pertussis IgG levels <20 FDA-U/mL are negative and >30 FDA-U/mL are positive. For GMT calculation, all negative values were assumed to be zero.

### 2.6. Safety Assessment

The subjects were closely monitored for 15 minutes following each dose. They recorded all adverse events in a diary card. Medical history was asked, and physical examination was done on each visit.

### 2.7. Statistics

Age was expressed in mean, standard deviation (SD) and median. Gender was expressed in percentages. Proportions of subjects with seroprotection for diphtheria and tetanus before and after vaccination were calculated and were compared by McNemar test. Percentages of subjects having baseline seronegative antipertussis IgG antibody titres were calculated. GMTs of anti-D and anti-T were calculated. “Paired *t*-test” was used to compare pre- and postvaccination GMTs. Incidence of adverse reactions was expressed in percentages.

## 3. Results

Total 62 subjects were screened and enrolled. The baseline blood samples were collected in all the subjects. Three subjects were lost to followup, while one missed one of the doses. The immunogenicity was assessed in 58 subjects. All the subjects were male. Mean age was 45 years (±7.7 years), while median was 43.5 years.

At baseline, 12% of the subjects did not have adequate protection against diphtheria (<0.1 IU/mL). 88% had safe protection (≥0.1 IU/mL) but only 9% had long-term protection (≥1 IU/mL). For tetanus, all of them had sufficient protection (≥0.1 IU/mL), but only 74% had long-term protection (≥1 IU/mL). For pertussis, 21% were seronegative, while 19% had borderline levels.

After receiving three doses of Td vaccine, the proportion of seroprotection changed significantly. All the subjects achieved safe protection, while 87% reached long-term protection for diphtheria (*P* < 0.02). For tetanus, 97% of the subjects attained long-term seroprotection. The change was significant. GMTs of both antibodies also increased significantly (Table 1).

In local reactogenicity, pain (26.34%, 95% CI 20.54–33.11) and swelling (1.07%, 95% CI 0.30 3.84) at injection site were reported. All of them were mild, lasted for 1–3 days, and resolved without any sequelae. Fever (<39°C) was reported in 2.69% (95% CI 1.15–6.14) of subjects. All the cases were with the first dose. Duration of fever was 1-2 days and resolved without any sequelae.

## 4. Discussion

In our study, 88% adult population was adequately protected against diphtheria, but only 9% had long-term protection. Short-term protection for tetanus was 100%, most probably because of frequent TT boosters. But here also, only 74% had long-term protection.

A study in Delhi among a random sample of healthy adults reported that 53% of adults were unprotected; 22% were seen to have only a basic protection against diphtheria; 25% were protected against both diseases; 47% were susceptible to tetanus [17]. Both the studies clearly demonstrate a need for Td vaccination in Indian adults, especially those who were never immunized.

Total 40% subjects were not adequately protected against pertussis. More than 50% of them had absolutely no seroprotection. This is a cause of concern and needs to be confirmed in larger studies. The developed countries have already seen a rise in the cases in the adolescent and the adult age group [18, 19]. Outbreaks have been reported among children and adults in countries such as Afghanistan, Israel, and Taiwan (Taipei) [20–22]. Though data from India is not available, it is quite likely that pertussis in adults may be a problem there also. There is definitely a need for larger serosurveys among adults as also studies defining the disease burden.

The study also demonstrated that Td vaccine in three doses induces an adequate immune response against diphtheria and pertussis in the unvaccinated adults. The study also demonstrated the safety and tolerability of the vaccine. The results are also in line with other studies on Td vaccine [16].

Despite certain limitations (the study was not community based, women were not represented, and small sample size), the study indicates that there is a definite need of Td vaccination in adult Indian population who did not receive primary immunisation with three doses of diphtheria-containing vaccines. Td vaccine of SIIL, when given in three doses in adults of 30–65 years of age, is immunogenic and safe. Susceptibility to diphtheria infection increases with increase in age. Larger studies should be undertaken in Indian adult population to determine prevalence of pertussis.

## Figures and Tables

**Table 1:** Comparison of long-term seroprotection and GMT with 95% CI.

Antigen		Prevaccination	Post-vaccination
Diphtheria	Long-term seroprotection	8.62 (3.74–18.64)	87.93 (76.6–95.1)
	GMT (IU/mL)	0.36 (0.28–0.47)	2.17 (1.66–2.83)
Tetanus	Long-term seroprotection	74.14 (60.9–84.8)	96.55 (87.9–99.7)
	GMT (IU/mL)	1.13 (1.01–1.27)	2.79 (2.21–3.52)

*All figures in parenthesis for Long-term seroprotection as well as GMT contains 95% CI.
